# Supplementary figures and images for: Clinical manifestations, associated risk factors and treatment outcomes of Chronic Pulmonary Aspergillosis (CPA): Experiences from a tertiary care hospital in Lahore, Pakistan
Source: PLoS One. 2021 Nov 12;16(11):e0259766. doi: 10.1371/journal.pone.0259766 (PMC8589179; doi:10.1371/journal.pone.0259766)

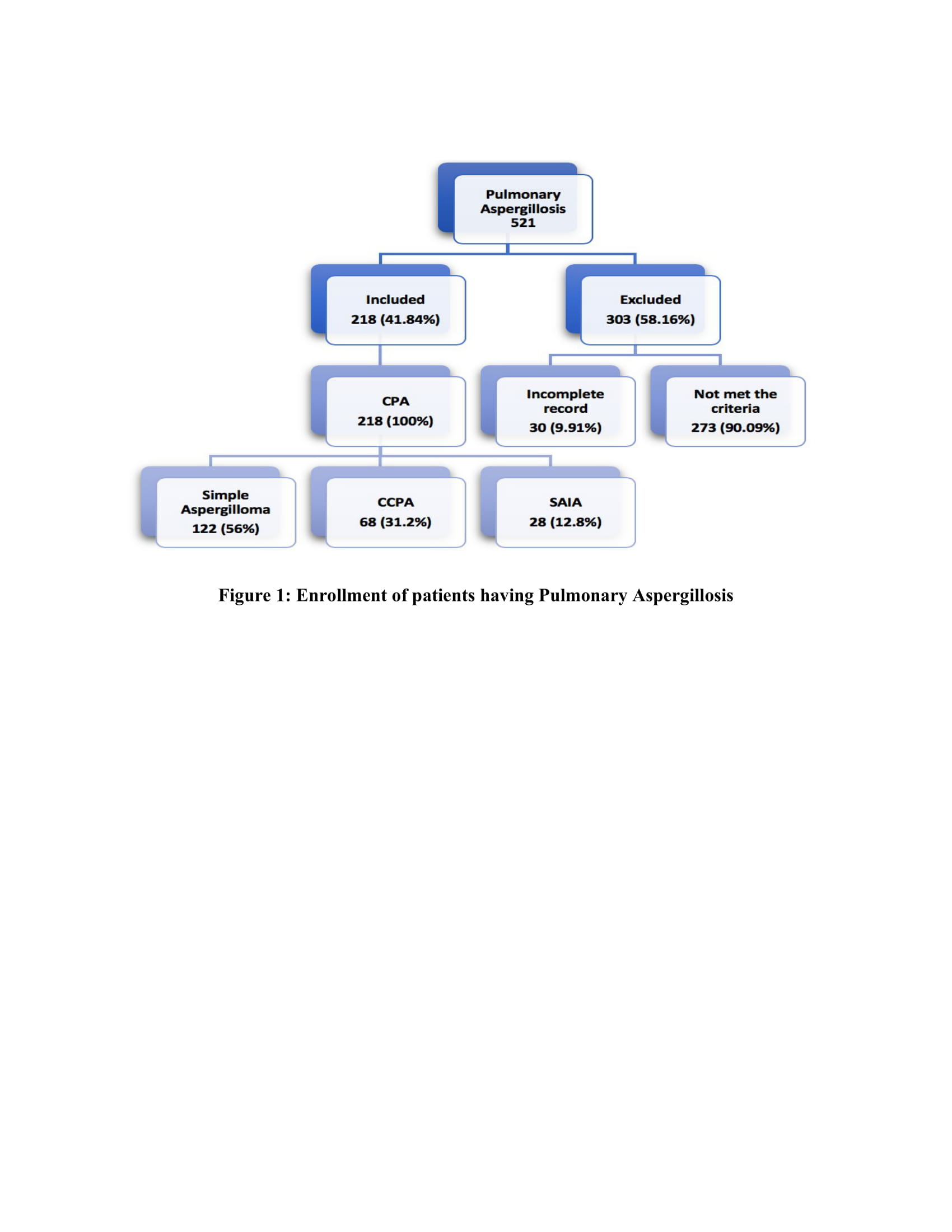

Supplement: S1 Fig — (TIF) [file pone.0259766.s001.tif]
