# Supplementary material for: Clinical manifestations, associated risk factors and treatment outcomes of Chronic Pulmonary Aspergillosis (CPA): Experiences from a tertiary care hospital in Lahore, Pakistan
Source: PLoS One. 2021 Nov 12;16(11):e0259766. doi: 10.1371/journal.pone.0259766 (PMC8589179; doi:10.1371/journal.pone.0259766)
Supplement: Attachment — Submitted filename: Response to Reviewers.docx [file pone.0259766.s003.docx]

**Point-by-Point response to reviewers.**

**Respected Editor,**

We have received revisions for our submitted manuscript. We have hereby addressed all the concerns of the reviewers and all the suggestions made by them have been duly acknowledged. We are hopeful that the concerns of all the reviewers will be satisfied by revised version of the manuscript. We have attached point-by-point response to the concerns of each reviewer and the revised version of manuscript has been highlighted in order to make the changes evident. Please let us know if there are any other suggestions or if any further changes are required.

We are thankful to the editor and the reviewers for their time and efforts in putting their valuable suggestions and recommendations to make this manuscript more scientifically elegant and technically sound.

**Response to the Academic Editor:**

1. Please ensure that your manuscript meets PLOS ONE's style requirements, including those for file naming. The PLOS ONE style templates can be found at

https://journals.plos.org/plosone/s/file?id=wjVg/PLOSOne_formatting_sample_main_body.pdf and

<https://journals.plos.org/plosone/s/file?id=ba62/PLOSOne_formatting_sample_title_authors_affiliations.pdf>

**Response: The revised manuscript has followed the direction.**

2. We suggest you thoroughly copyedit your manuscript for language usage, spelling, and grammar. If you do not know anyone who can help you do this, you may wish to consider employing a professional scientific editing service.

**Response: The revised manuscript has followed the direction. The manuscript edited for language usage, spellings and grammar by Dr. Tauqeer Hussain Mallhi (Assistant Professor, Department of Clinical Pharmacy, College of Pharmacy, Jouf University, Sakaka, Al-Jouf, Saudi Arabia.**

3. Thank you for including your statement regarding patient consent from living patients. In your ethics statement in the manuscript and in the online submission form, please clarify whether any patients were unable to provide written informed consent regarding the use of the clinical records e.g. because they were deceased; if so, please state whether the IRB or ethics committee waived the requirement for informed consent from these patients.

**Response: The IRB of the Hospital waived the requirement for informed consent from all those patients who were Critical or Deceased.**

4. In the ethics statement in the manuscript and in the online submission form, please provide additional information about the patient records/samples used in your retrospective study, including: a) whether all data were fully anonymized before you accessed them; b) the date range (month and year) during which patients' medical records/samples were accessed.

**Response: a) All the data were fully anonymous. b) The patients who were enrolled for the treatment at the facility from January 1^st^ 2017 to December 30^th^ 2019 were included in this study.**

5. PLOS requires an ORCID iD for the corresponding author in Editorial Manager on papers submitted after December 6th, 2016. Please ensure that you have an ORCID iD and that it is validated in Editorial Manager. To do this, go to ‘Update my Information’ (in the upper left-hand corner of the main menu), and click on the Fetch/Validate link next to the ORCID field. This will take you to the ORCID site and allow you to create a new iD or authenticate a pre-existing iD in Editorial Manager. Please see the following video for instructions on linking an ORCID iD to your Editorial Manager account: <https://www.youtube.com/watch?v=_xcclfuvtxQ>

**Response: ORCID ID has been created and linked to Editorial Manager Profile.**

**Reviewer 1: Reviewer Comments**

**Reviewer:** This is a fairly straightforward retrospective study of outcomes in pulmonary aspergillosis. Overall it provides helpful granular information regarding risk of mortality and complications for various types of pulmonary aspergillosis and as such is of value to the literature.

**Response:** Respected reviewer, we are very thankful to you for acknowledging our work. We really appreciate your interest in the draft and welcome any further concerns to address.

**Query 1:** There are many grammatical and spelling errors and the document would benefit from an edit by a native english speaker.

**Response 1:** Respected reviewer, all the errors have been corrected and the manuscript has been reviewed and revised by a native English speaker as recommended. We expect that the revised manuscript will satisfy this concern.

**Query 2:** Mortality is discussed but I don't see any discussion of the average duration of follow up for cases. Mortality is 100% if the follow up period is long enough.

**Response 2:** Respected reviewer, we have incorporated the median duration of follow up in Table 3 as well as in the discussion under heading: Antifungal therapy and outcomes.

**Query 3:** I don't know if it adds anything to say that longer LOS and ICU admission predict mortality as hospitalization and ICU admission tend to proceed mortality and are outcomes themselves.

**Response 3:** Respected reviewer, we agree that hospitalization and ICU admission are also the outcomes. We have now analyzed and mentioned the significant factors leading to longer LOS and ICU admission in discussion under the heading: Factors associated with mortality. In our study, the recovery rates in patients with prolonged LOS and ICU admission were very low. In short, the non-survivors had a longer ICU stay and greater Mean LOS. That’s why we have listed both the longer LOS and ICU admission as the significant factors that lead towards our focused outcome, i.e., Mortality itself. However, we welcome further suggestions regarding this very aspect.

Respected reviewer,

We have tried our best to improve the manuscript and have made some changes in the manuscript. In this point-by-point response document, we did not list all the changes, but the changes are marked in the revised version. We are hopeful that this revision will meet with approval.

**Reviewer 2: Reviewer Comments**

**Reviewer:** The manuscript by Akram et al descibes clinical characteristics of a cohort of patients with pulmonary aspergillosis. The strenght of the study is the large number of patients with postTB aspergilloma. However, the topic has been extensively reviewed in the last years and the manuscript does not add information to current knowledge. The english academic language needs some corrections.

**Response:** Respected reviewer, we are thankful to you for acknowledging the strengths of our study. As the matter of fact, Pakistan is ranked among the countries with a significant burden of tuberculosis whereas the data available regarding the various manifestations of CPA alone and its characteristics as a sequel to pulmonary tuberculosis, risk factors, treatment outcomes and prognosis in patients with collective presence of TB and CPA is scarce. In the revised edition, we have focused greatly on the impact on prognosis in patients of CPA with co-existing pulmonary tuberculosis as well. I expect that the revised edition of manuscript and the fact that very scarce data is available regarding this very aspect in Pakistan’s scenario, will satisfy your concern. Furthermore, the mistakes in English academic language have been corrected and the manuscript has been extensively reviewed and revised by a native English speaker.

Respected reviewer,

We have tried our best to improve the manuscript and have made some changes in the manuscript. In this point-by-point response document, we did not list all the changes, but changes are marked in the revised version. We are hopeful that this revision will meet with approval.

**Reviewer 3: Reviewer Comments**

Reviewer: The study investigated the clinical manifestations, associated risk factors and treatment outcomes of chronic pulmonary aspergillosis in a tertiary care hospital in Pakistan. It’s important. However, there are several issues in the study.

Response: Respected reviewer, we are very thankful to you for acknowledging the significance of our study. I expect that the revised version will satisfy all the points raised.

**Query 1:** The dosage and course of steroid, and respiratory complications (Table 4) were not clearly described in the study.

**Response 1:** Respected reviewer, the dosage and course of steroid has been incorporated in the discussion under heading: Underlying Conditions. Details about respiratory complications were present in the Table 3 and also in discussion under the heading: Antifungal therapy and outcomes. Moreover, details of respiratory complications have now been incorporated in the Table 5 and their association with mortality has also been analyzed. We expect that the revised edition will satisfy the concerns.

**Query 2:** Age distribution, treatment choice, and the impact on prognosis in patients with tuberculosis is not well analyzed.

**Response 2:** Respected reviewer, age distribution, treatment choice and the impact on prognosis in patients with tuberculosis has now been incorporated as a separate heading: Impact of prognosis in patients with TB. Where the antifungal treatment in patients with co-existing TB has been clarified and its outcomes have been discussed. We are hopeful that the revised manuscript will satisfy this point raised.

**Query 3:** Moreover, many factors such as underlying conditions and treatment medications may also impact the alarming mortality rate. More detailed analysis is needed.

**Response 3:** Respected reviewer, a detailed analysis about the impact of underlying conditions (such as diabetes mellitus, hematological malignancies, chronic kidney disease, chronic liver disease, steroid use, etc.) has been performed and in the revised manuscript, a separate portion in Table 5 with title: Underlying conditions associated with mortality, has been created to clarify things. Furthermore, the treatment choices have also been included in the univariate analysis to unveil their impact on mortality and the results have been mentioned in Table 5. We expect that the revised edition will satisfy your concerns.

Respected reviewer,

We have tried our best to improve the manuscript and have made some changes in the manuscript. In this point-by-point response document, we did not list all the changes, but the changes are marked in the revised version. We are hopeful that this revision will meet with approval.
