# Supplementary material for: Clinical manifestations, associated risk factors and treatment outcomes of Chronic Pulmonary Aspergillosis (CPA): Experiences from a tertiary care hospital in Lahore, Pakistan
Source: PLoS One. 2021 Nov 12;16(11):e0259766. doi: 10.1371/journal.pone.0259766 (PMC8589179; doi:10.1371/journal.pone.0259766)
Supplement: Attachment — Submitted filename: Response to Reviewers.docx [file pone.0259766.s004.docx]

**Point-by-Point Response.**

**Respected Editor,**

We have received revision for our submitted manuscript. We have hereby addressed all the concerns of the reviewer and all the suggestions made by the reviewer have been duly acknowledged. We are hopeful that the concerns of the reviewer will be satisfied by revised version of the manuscript. We have attached point-by-point response to the concerns of the reviewer and the revised version of manuscript has been highlighted in order to make the changes evident. Please let us know if there are any other suggestions or if any further changes are required.

We are thankful to the editor and the reviewers for their time and efforts in putting their valuable suggestions and recommendations to make this manuscript more scientifically elegant and technically sound.

**Journal Requirements:**

**1.** Please review your reference list to ensure that it is complete and correct. If you have cited papers that have been retracted, please include the rationale for doing so in the manuscript text, or remove these references and replace them with relevant current references. **Any changes to the reference list should be mentioned in the rebuttal letter that accompanies your revised manuscript.** If you need to cite a retracted article, indicate the article’s retracted status in the References list and also include a citation and full reference for the retraction notice.

**Response:** There is no retracted reference in our article. We did some changes to the reference list in the previous revision. Following are the new references that were added:

| **Number in References** | **References Added** |
| --- | --- |
| **25.** | Kosmidis C, Denning DW. The clinical spectrum of pulmonary aspergillosis. Thorax. 2015;70(3):270-7. |
| **26.** | Ader F, Nseir S, Le Berre R, Leroy S, Tillie-Leblond I, Marquette C, et al. Invasive pulmonary aspergillosis in chronic obstructive pulmonary disease: an emerging fungal pathogen. Clinical Microbiology and Infection. 2005;11(6):427-9. |
| **36.** | Uzunhan Y, Nunes H, Jeny F, Lacroix M, Brun S, Brillet P-Y, et al. Chronic pulmonary aspergillosis complicating sarcoidosis. European Respiratory Journal. 2017;49(6). |

Apart from that, in this revision, a duplicated reference has been removed. The reference present at number 47 in the previous version, was also present at number 12. Below are the details of that particular reference:

| **Number in previous version.** | **Reference Removed** |
| --- | --- |
| **47.** | Iqbal N, Irfan M, Zubairi ABS, Jabeen K, Awan S, Khan JA. Clinical manifestations and outcomes of pulmonary aspergillosis: experience from Pakistan. BMJ open respiratory research. 2016;3(1). |

**2.** Please ensure that you refer to Table 4 in your text as, if accepted, production will need this reference to link the reader to the Table.

**Response: The revised manuscript has followed the direction.**

**3.** Please upload a copy of Supporting information Figure S1 which you refer to in your text on line 586.

**Response: The revised manuscript has followed the direction.**

**Reviewer 2: Reviewer Comments**

**Reviewer:** The manuscript has been greatly improved. The description of statistical analysis needs further clarification regarding the multiple regression analysis and how parameters were selected. In the tables it should be clarified whether range, IQR or SD is presented.Theere are still a number of spelling errors.

**Response:** Respected reviewer, we are pleased to know that you have seen great improvements in our revised manuscript. Your concern towards our manuscript is really appreciated and we are thankful that you are taking pains to recommend further improvements.

**Concern 1:** The description of statistical analysis needs further clarification regarding the multiple regression analysis and how parameters were selected.

**Response 1:** Respected reviewer, prior to performing the multivariate analysis, univariate analyses of the independent variables with the outcomes (that were stable/recovered, Deteriorated, and Mortality) were conducted. For the univariate analysis, all the parameters/variables that were clinically and biologically plausible, and all those indicated by previous literature, were selected. The variables reaching significance on univariate analyses, i.e., p ≤ 0.05, were selected for multinomial logistic regression analysis, in order to determine the independent factors associated with mortality. Furthermore, in regression output, the redundant parameters were excluded from the model. The results were presented in tables as p-value and odds ratio, along with 95% confidence intervals. For your convenience, we have highlighted the text in methodology, in which we have mentioned that how the variables were selected.

**Concern 2:** In the tables it should be clarified whether range, IQR or SD is presented.

**Response 2:** Respected reviewer, the mentioned aspect has been clarified and highlighted in tables.

**Concern 3:** Theere are still a number of spelling errors.

**Response 3:** Respected reviewer, all the spelling errors have been corrected and the manuscript has been subjected to a review by a native speaker. We expect that the revised version will satisfy this concern.

Respected reviewer,

We have tried our best to improve the manuscript and have made and highlighted the recommended changes in the manuscript. In this point-by-point response document, we did not list all the changes, but the changes are marked in the revised version. We are hopeful that this revision will meet with approval.
