# Supplementary material for: Clinical manifestations, associated risk factors and treatment outcomes of Chronic Pulmonary Aspergillosis (CPA): Experiences from a tertiary care hospital in Lahore, Pakistan
Source: PLoS One. 2021 Nov 12;16(11):e0259766. doi: 10.1371/journal.pone.0259766 (PMC8589179; doi:10.1371/journal.pone.0259766)
Supplement: Attachment — Submitted filename: Response to Reviewers.docx [file pone.0259766.s005.docx]

**Point-by-Point Response.**

**Respected Editor,**

We have received further revision for our submitted manuscript. The concerns of the reviewers have been duly acknowledged. We are hopeful that the concerns of the reviewers will be satisfied by this version of the manuscript. We have attached point-by-point response to the concerns of the reviewers and the revised version of manuscript has been highlighted in order to make the changes evident.

We are greatly thankful to the editor and the reviewers for their time and efforts in putting their valuable suggestions and recommendations to make this manuscript scientifically elegant, technically sound and comprehendible.

**Reviewer Comments**

**Reviewer 1:** Explanation of the statistical analyses has greatly improved. The document is still somewhat difficult to read and it would benefit from a once over by a native english speaking editor, but I think the data and conclusions are sound.

**Response:** Respected reviewer, we are pleased to know that you are satisfied with the explanation of statistical analyses. The manuscript has been subjected to review by a native English-speaking editor and Proofreading certificate is attached. We are hopeful that the revised version will satisfy this concern.

Respected reviewer,

We have tried our best to improve the manuscript by subjecting it to an extensive review by a native English speaking editor. We are hopeful that this revision will meet with approval.

**Reviewer 2:** The manuscript has been greatly improved. Comments have been addressed. However the academic language needs revision.

**Response:** Respected reviewer, we are pleased to know that you have seen great improvements in the manuscript. The manuscript has been subjected to review by a native English-speaking editor and Proofreading certificate is attached. We are hopeful that the revised version will satisfy this concern.

Respected reviewer,

We have tried our best to improve the manuscript by subjecting it to an extensive review by a native English-speaking editor. We are hopeful that this revision will meet with approval.
